# Combining Surface Templating and Confinement for Controlling Pharmaceutical Crystallization

**DOI:** 10.3390/pharmaceutics12100995

**Published:** 2020-10-20

**Authors:** Manali Banerjee, Blair Brettmann

**Affiliations:** 1 School of Materials Science and Engineering, Georgia Institute of Technology, Atlanta, GA 30332, USA; bmanalib@gatech.edu; 2 School of Chemical and Biomolecular Engineering, Georgia Institute of Technology, Atlanta, GA 30332, USA

**Keywords:** crystallization, surface templating, confinement, combined crystallization, API nucleation

## Abstract

Poor water solubility is one of the major challenges to the development of oral dosage forms containing active pharmaceutical ingredients (APIs). Polymorphism in APIs leads to crystals with different surface wettabilities and free energies, which can lead to different dissolution properties. Crystal size and habit further contribute to this variability. An important focus in pharmaceutical research has been on controlling the drug form to improve the solubility and thus bioavailability of APIs. In this regard, heterogeneous crystallization on surfaces and crystallization under confinement have become prominent forms of controlling polymorphism and drug crystal size and habits; however there has not been a thorough review into the emerging field of combining these approaches to control crystallization. This tutorial-style review addresses the major advances that have been made in controlling API forms using combined crystallization methods. By designing templates that not only control the surface functionality but also enable confinement of particles within a porous structure, these combined systems have the potential to provide better control over drug polymorph formation and crystal size and habit. This review further provides a perspective on the future of using a combined crystallization approach and suggests that combining surface templating with confinement provides the advantage of both techniques to rationally design systems for API nucleation.

## 1. Introduction

Oral drug delivery is the most common and preferred form of drug administration into the body, especially for small molecule active pharmaceutical ingredients (APIs). On account of high patient compliance, affordability, and acceptance, tablets and pills are the leading route of drug delivery with recent studies showing that 52% of new drugs approved by the U.S. Food and Drug Administration (FDA) in 2018 were oral dosage forms [1]. Oral dosage forms have wide ranging applications from pain medication to HIV treatment; however, certain disadvantages, including poor drug solubility, bioavailability, and stability, lead to limitations with their therapeutic abilities [2,3,4]. This results in ~40% of newly discovered APIs failing during development, especially due to poor water solubility [5,6,7].

APIs often have a large number of functional groups in their chemical structure enabling them to pack into several different crystal structures or polymorphs. Polymorphic materials are those with the same chemical composition that can crystallize into different lattice structures or conformations [3,8]. Predicting and controlling the API polymorph that forms is extremely challenging due to the large number of functional groups and the variety of conformations each molecule can take in space. Each of the polymorphs have unique physical and chemical properties including shape, purity, and free energy [3,9,10], which can lead to vastly different behavior in terms of stability, solubility, dosage, and exposure limits [10,11,12].

**Figure 1:**
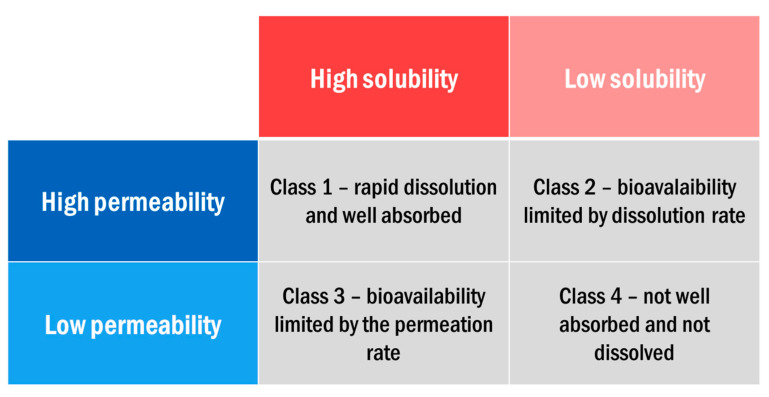
BCS classification system for orally administered drugs (modified and reprinted with permission from [14], Elsevier, 2012).

An important factor in determining the effectiveness of orally delivered drugs is the bioavailability, which is defined as the rate and extent to which an administered drug is absorbed and becomes available to the site of drug action [3,13]. This can be thought of in terms of two parameters: solubility and permeability. Solubility refers to the volume of water required to dissolve the highest dose of the drug at physiological pH ranges and permeability refers to the absorption of the drug into the blood circulation. The biopharmaceutical classification system (BCS) divides orally delivered drugs into four categories based on these two parameters (Figure 1).

Frequently, for class 2 and class 4 drugs (low solubility), the required clinical dosage of a drug is insoluble in physiological amounts of gastrointestinal fluid, rendering the drug inaccessible to the body [3]. According to the U.S. Pharmacopoeia, solubilities below 10 mg/mL are considered sparingly soluble and under 0.1 mg/mL are practically insoluble [3,15,16]. To dissolve some of these drugs such as piroxicam, a pain medication, over 2 L of GI fluid would be required [13]. APIs with these low solubilities often pass their absorption site within the body (such as the stomach or intestine) before complete dissolution of the drug crystal [10,17,18].

Research into controlling the drug form in order to improve solubility and bioavailability is an important focus in the pharmaceutical field. As such, there has been a steady focus on customizing the form of APIs in order to optimize the dissolution and bioavailability. Heterogeneous crystallization from surfaces has been one of the most controllable methods for tailoring formation of desired API polymorphs. A more recent approach has been crystallization under confinement in order to limit the critical size of a nucleus, which controls the final drug crystal form. Salt and cocrystal formation are additional routes to manipulating the physical form of a drug to increase solubility [19]. Forming an ionizable salt of a drug compound enables easier aqueous diffusion and increases solubility and while this is a commonly used technique in the pharmaceutical industry, it is sometimes not possible to obtain the salt form of an API [19,20]. Cocrystals are crystalline assemblies of multiple molecular compounds that form a single phase. Combining an API with a coformer and forming a cocrystal can change the underlying crystal structure of the API and increase its bioavailability [21]. Several authors have reviewed salts [19,22,23,24] and cocrystals [19,20,21,25,26] of APIs but these crystal systems are not discussed further here since this review is focused on crystallization on/in templates to control polymorphism.

Additionally, while some reviews have presented the current state of studies relating to heterogeneous crystallization from surfaces [27,28] and crystallization within nanopores [29,30,31], this review focuses on studies using a combination of these methods to tailor the drug form. We first introduce polymorphism in Section 2, then discuss heterogeneous crystallization and crystallization under confinement in Section 3 and Section 4 to provide a strong context for Section 5, which reviews the state of the art on combined approaches to controlling crystallization. We finish with a perspective on the future in Section 6, suggesting that a combined crystallization approach provides more levers for control over the rational design of API forms in order to obtain drug products with the highest bioavailability and best long-term stability.

## 2. Polymorphism in Pharmaceuticals

**Figure 2:**
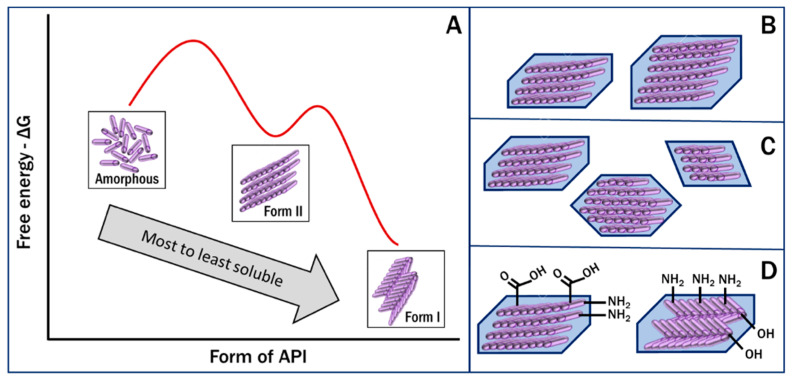
Key factors affecting the dissolution rate of pharmaceuticals. (**A**). free energy of the API form, (**B**). crystal size, (**C**). crystal habit, and (**D**). surface functional groups.

The exposed surface of API particles is determined by the form of the drug, which can be different crystalline polymorphs or a disordered amorphous form [9,10,32]. The functional groups present on the surface and their configurations determine the ability of the drug molecules in the crystal to form intermolecular bonds with each other and with the GI fluid. These interactions then control the wetting and impact the dissolution of a solid drug particle [11,33]. The free energy of a crystal determines its inherent stability and the amount of energy needed to separate the lattice and release the molecules into the solvent [34,35]. Additionally, the size and shape of the crystal determine the surface area-to-volume ratio of a particle available for these interactions and bond formations to occur. Thus, the polymorph properties of surface wettability, free energy and particle shape, in addition to a property set primarily by processing, the size, are the key drivers for the rate of dissolution and solubility achievable physiologically (Figure 2). The interest in pharmaceutical polymorphism arises from these property differences, which need to be understood in the development of a specific drug product.

The lowest energy polymorph of an API is typically the most easily obtained and the most thermodynamically stable. The stability makes this form desirable to manufacturers since it is least likely to change during scale up and processing [35,36]. However, it could show very low solubility and bioavailability, making it therapeutically ineffective. An example is chloramphenicol palmitate (CAP), with polymorphs A and B. While A is the thermodynamically stable form, it is therapeutically inactive. Form B is a higher energy form, so it dissolves more easily in water, raising its bioavailability [37,38,39]. Aguiar et al. showed that the peak blood serum level of CAP increases linearly as the percentage of form B is increased from ~3 μg/mL (for all form A) to 22 μg/mL (for 100% form B) [39].

### 2.1. The Amorphous Form

Though the amorphous form is outside the scope of this review, it is essential to mention in any discussion of solid drug forms. While crystalline materials have three-dimensional long-range order, amorphous materials exist with more random packing, resulting in higher internal energy [40]. Amorphous forms of pharmaceuticals have, therefore, been shown to have improved solubility and higher bioavailability as a result of being a highly energetic solid material, indicating that less energy is required to transfer molecules from the drug to the solution [41,42]. Owing to this, amorphous APIs typically have a higher dissolution rate, which has been documented in several studies [16,41]. For example, Aucamp et al. showed that amorphous azithromycin (AZM), prepared by quench cooling from the melt state, has a much higher solubility in distilled water than crystalline and dihydrate forms [43], and a study by Dhumal et al. showed that crystalline cefuroxime axetil (CEF), an orally delivered antibiotic, only achieved 45% dissolution over the course of a 150 min experiment, while amorphous CEF, prepared from ultrasonic treatment, achieved 100% dissolution within 60 min [44]. Despite their high solubility, the use of the amorphous form is limited due to its poor stability and a tendency to crystallize over time [16,41,42].

Typically, commercially available amorphous forms of APIs are prepared in conjunction with polymeric excipients, such as polyvinylpyrrolidone (PVP) and hydroxypropyl methylcellulose (HPMC), in order to prevent crystallization and increase bioavailability [15,45,46,47]. The amorphous form may be produced in a combined confinement and surface templating precipitation process, similar to the crystal forms that are the focus of this review, but it is more desirable to have molecular level mixing of the amorphous form with the polymer excipient for stability [48,49,50,51], so this is not a focus of the combined confinement and surface templating studies.

### 2.2. Polymorph Stability and Metastable Forms

Concerns with the stability of drug forms is not limited to the amorphous form. API polymorphs can also undergo interconversion and this is an important factor to consider during drug development. Metastable polymorphs of a drug can often form during the crystallization process due to kinetic factors. These are transitional states that are lower in energy than a supersaturated solution but are not the most thermodynamically stable form. Metastable polymorphs have higher solubilities than the thermodynamically favored state but face the same stability issue as amorphous forms [28,34]. Chloramphenicol palmitate (CAP) is an example of this phenomenon. While form B of CAP has higher solubility, it shows lower thermodynamic stability, leading to conversion to form A over time [34,37,38]. Similar to amorphous drugs, polymeric additives and other excipients can be added to metastable polymorphs in order to stabilize these drug forms. Telford et al. showed that a metastable form III of acetaminophen (ACT), which is typically only crystallized in specialized glass capillaries, was stabilized using lactose as an excipient. This was achievable due to the favorable interactions between the lactose molecules and the ACT hydroxyl (OH) groups [52,53].

In addition to conversion, different polymorphs of a drug can also exhibit different degradation behavior with exposure to environmental factors, such as moisture, temperature, and light [11]. Carbamazepine (CBZ), an anticonvulsant drug, exists as several different polymorphs and has been highly studied for several decades. Matsuda et al. showed that the decay of CBZ form II in the presence of UV-light was significantly faster than that of forms I and III [54]. This was attributed to the highest hygroscopicity of form II resulting in faster surface decomposition [54,55].

The properties of solid drug forms can differ vastly and the form of a drug is highly dependent on the crystallization conditions, including solvent, temperature, concentration of drug, and cooling rate [4,34,56,57,58]. As such, control over these conditions during API formulation and production is essential for obtaining consistent desired drug properties.

## 3. Individual Approaches to Polymorph Control

Crystallization consists of two steps: nucleation and growth. Nucleation is the formation of a small particle out of a fully dissolved solution and growth occurs as additional molecules from the solution attach onto the nuclei. Nucleation controls the type of crystal structure obtained and the size and shape, or crystal habit, is affected by the environmental conditions during the growth process [59,60]. Both the crystal structure and the size and shape of a drug crystal control the surface area and the amount of hydrophilic facets available for wettability and dissolution of the drug, therefore greatly influencing solubility and bioavailability [34,61,62]. While growth plays an important role in the final solubility, we focus primarily on nucleation, when the crystal polymorph is determined.

**Figure 3:**
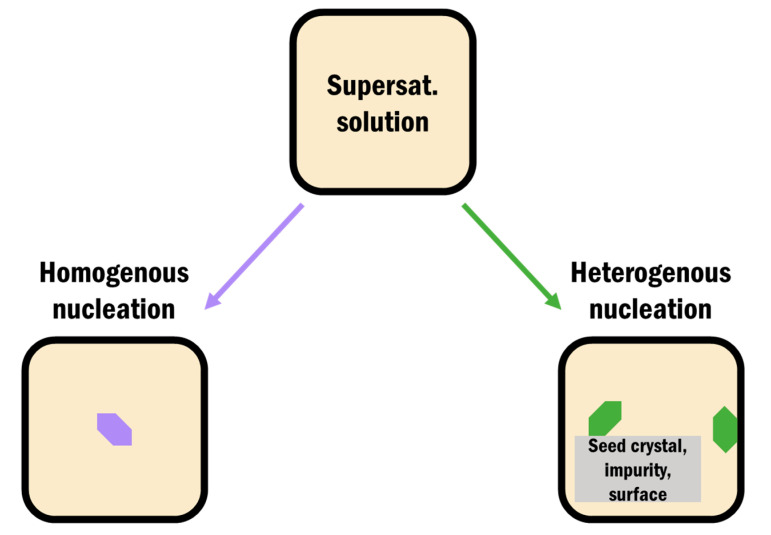
Schematic representation of homogeneous nucleation directly out of a supersaturated solution and heterogeneous nucleation from a container surface, added impurity, or seed crystal.

Nucleation in pharmaceutical crystallization falls into two main categories: homogeneous and heterogeneous. Homogeneous nucleation is the formation of a solid nucleus spontaneously in solution without any preferred nucleation site. Heterogeneous nucleation is the formation of a new nucleus directly onto another surface (Figure 3) [56,63]. Nuclei formation is influenced by contributions from intermolecular interactions, flexible molecular conformations, and solvent dynamics and the driving force for both of these mechanisms is a decrease in the free energy of the system [29]. There are a variety of crystallization techniques currently used in industry to control the nucleus formation and thus crystal properties, such as high-pressure homogenization and supercritical fluid crystallization [2,32]; however, many of them can lead to production of impure and undesired polymorphs since crystallization is a highly sensitive process [34]. For example, when batch cooling of glycine was done at 20–30 °C with ultrasound treatment, almost 90% purity of the α form was achieved. The purity dropped to around 60% when a slightly higher temperature of 40–50 °C was used, with the same ultrasound treatment [64]. Similarly, a slow freezing rate of mannitol produced a mixture of polymorphs α and β, while a faster freezing rate resulted in the γ form [65]. The form of a drug obtained during crystallization depends on several factors including temperature, freezing rate, solvent, and concentration [4,34,65,66]. In order to minimize some of these effects, there has been a shift towards more controllable drug crystal engineering processes such as heterogeneous crystallization onto tailored surfaces and confined crystallization within pores [27,31,67,68,69].

### 3.1. Heterogeneous Crystallization on Surfaces

Heterogeneous crystallization involves nucleation onto an existing surface, which decreases the effects of molecular conformations and the solvent, while providing a surface as a template [70,71,72]. This has historically been used through the practice of “seeding” the crystallization process, where pre-formed crystals of the desired polymorphs are introduced to the supersaturated drug solution so that additional nuclei orient into the same crystal structure [73,74]. However, this seeding method is not sufficient for new challenges in production of thermodynamically unfavorable polymorphs, as it requires a significant quantity of pre-formed crystals of the same polymorph, which could be difficult and costly to produce. Additionally, with metastable polymorphs, there is also the chance of interconversion of the seeded crystals to more stable, but undesired, forms of a drug. Nichols et al. used seeds to crystallize larger quantities of the metastable form II of ACT; however, when left in solution the prismatic form II crystals underwent redissolution and crystallized as plate-like form I crystals [75]. Seeding also does not completely eliminate the formation of undesired polymorphs from the supersaturated bulk solution [69,76]. This has resulted in the need for new crystallization approaches involving heterogeneous crystallization onto surfaces that are not identical to the APIs, but where the desired polymorph is more favorable than the most stable form [28,67,77,78].

**Figure 4:**
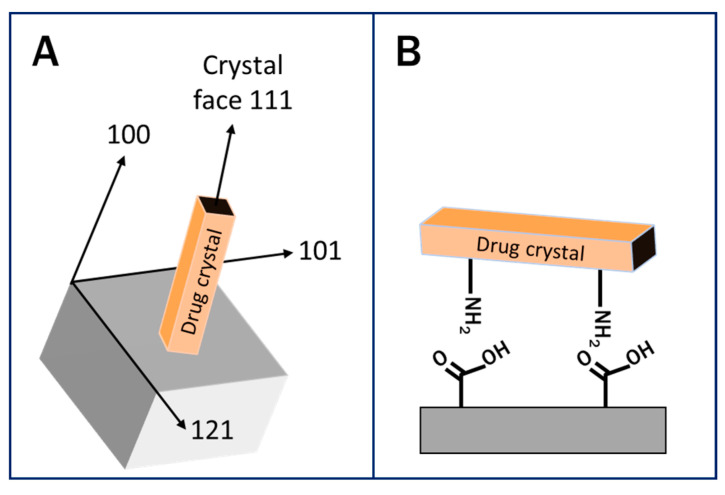
Parameters affecting heterogeneous crystallization: (**A**) Lattice matching and (**B**) Surface chemistry.

In designing surfaces for heterogeneous nucleation, the key parameters are epitaxial matching (lattice structure) [79,80,81] and the surface chemistry (physical interactions to control molecular orientation) (Figure 4) [63,82,83]. Additionally, having a lower area available for crystallization leads to higher supersaturation, which can be used to induce formation of desired nuclei. This is particularly important for crystallizing metastable polymorphs, as metastable phases form more readily at high supersaturations [28,84,85].

Epitaxial matching involves matching the lattice structure of a surface with that of the growing crystal and has been used by several groups to direct the growth of a specific crystal [80,86,87,88]. Mitchell et al. showed that by using cleaved surfaces of pimelic acid (PA) crystals, epitaxial matching could be used to form the yellow needle (YN) polymorph of 5-methyl-2-[(2-nitrophenyl)amino]-3-thiophene-carbonitrile (ROY). PA crystals present with (101) and (111) planes but the YN polymorph preferentially nucleates on the (101)_PA_ plane due to direct contact with the (001)_YN_ plane. This study showed that there was two-dimensional epitaxial matching between these YN crystals and the PA surfaces using atomic force microscopy and the exact orientation was [100]_YN_-[010]_PA_ and [010]_YN_-[101]_PA_ [80].

Another method for controlling nucleation is through the use of the surface’s chemistry. Specific functional groups on a pharmaceutical molecule can be targeted via the formation of hydrogen bonding or steric effects and used to direct the nucleation of a desired polymorph [63,83,89,90]. Hydrogen bonding between microcrystalline cellulose (MCC) and API crystals was used by Verma et al. to drive heterogeneous nucleation for several APIs including carbamazepine (CBZ), acetaminophen (ACT), and caffeine (CAF) [63]. Caridi et al. found that the stable form II of isonicotinamide (INA) is obtained without surface templating, but if crystallized in the presence of powdered anatase TiO2, INA forms polymorphs I and III, which are typically metastable due to interactions of the anatase oxygen atoms with NH_2_ groups of INA [91].

Several studies have also looked at self-assembled monolayers (SAMs) as templates for API polymorph crystallization. SAMs are highly ordered and can incorporate a wide range of functional groups, which can be used to readily alter the surface chemistry of a substrate [82,92,93,94]. This makes SAMs very versatile for controlling crystal growth and several groups have used these materials to direct polymorph growth [69,82,92,93,95]. Hiremath et al. used 3′-nitro-4-mercaptobiphenyl SAMs to preferentially orient the less stable orthorhombic crystals of 2-iodo-4-nitroaniline via I-NO_2_ interactions and van der Waals forces between the crystal and the SAM substrate [82]. Yang et al. prepared SAMs of 3-mercaptopropionic acid and nucleated the metastable form II of the drug mefenamic acid by forming hydrogen bonding interactions between the substrate and the -COOH groups of the drug crystals [93]. Similarly, Dressler et al. used SAMs to grow the less stable polymorphs of L-Glutamic acid (GLU). They used SAMs of l-2-amino-N-{-[2-(2-amino-3-phenyl-propionylamino)-ethyldisulfanyl]-ethyl}-3-phenyl-propionamide (LAAPP) to nucleate the metastable α-form of GLU and stabilize it so that no polymorphic transition occurred [95].

### 3.2. Confinement in Pores

Confinement of drug solutions in pores (or other spaces that are smaller than a few hundred nanometers) can induce crystallization by increasing the supersaturation in localized areas. This can lead to homogeneous nucleation within the pores or even heterogeneous nucleation from pore walls [29]. Critical nucleus size (r_crit_) depends on the polymorphic form and pores constrain the space available for nucleus formation, which can be used to direct the formation of certain drug polymorphs [53,96]. As noted previously, each polymorph has a characteristic free energy of formation (ΔG_cryst_) and while a certain polymorph may be more stable in the bulk, metastable forms can have a lower ΔG_cryst_ at r_crit_ leading to stabilization of the metastable form under confinement [31]. Confinement within pores also limits the amount of space available for the growth of a drug crystal, leading to smaller crystals, which have a higher surface area-to-volume ratio and thus a faster dissolution rate [30,32]. Variations in the pore sizes can also lead to different crystal habits due to interactions with the solvent or availability of/access to certain crystal faces [30,32,97,98].

Several studies have used confinement to direct the crystallization of specific polymorphs or even force the formation of the amorphous form of a drug and most of these have focused on using inorganic materials [29,53,96,97,99] or polymers [29,100,101] for drug confinement. It has been suggested that in order for crystal nucleation to take place, pore sizes need to be about 20 times the size of the critical nucleus. Recently, Dwyer et al. confirmed this for the case of fenofibrate (FEN), a drug used to lower cholesterol and triglycerides, with a critical nucleus of ~1.27 nm. They used nanoporous silica and showed that below a 12.7 nm pore size, amorphous FEN was formed, whereas above a 20.2 nm pore size, crystallization could occur and FEN form I was grown [97]. Glass membranes with different pore sizes were used by Ha et al. to crystallize anthranilic acid (AA), a water-soluble vitamin and metabolite. The smallest pore size was able to nucleate the metastable form II of AA, while larger pores revealed only form III of the drug. The preference for form II in the smaller pores was attributed to the smaller critical nucleus size [96]. Similarly, Beiner et al. used controlled pore glass to crystallize ACT and showed that using nanoconfinement, the metastable form III of ACT was crystallized and stabilized when the pore sizes were between 43 and 103 nm [53].

Crystallization control has also been achieved through confinement in environments other than pores. Lee et al. was able to change the crystal forms of glycine produced using gold islands covered with SAMs of 4-mercaptobenzoic acid with varying island dimensions [32]. Al-Ani et al. used an electrospray technique to confine ACT into nanodroplets, which resulted in the formation of the metastable polymorph II. They showed that using confinement, 98% of the nucleated crystals were ACT-II and 2% was ACT-I [102]. These studies show that confinement is promising for directing pharmaceutical polymorphism.

Additionally, a modeling approach can be used to study nucleating drug crystals under confinement in order to choose specific pore sizes to direct polymorphism. By determining the r_crit_ for different polymorphs, pore sizes can be chosen to induce or suppress specific API polymorphs or even amorphous forms of drugs [29,103]. Studies that combine computational modeling with experimental approaches to crystallization under confinement propels crystallization research towards rational design of nanoscale systems for crystallization.

## 4. Combining Surface Chemistry and Confinement for Pharmaceutical Crystallization

Surface nucleation and confinement within pores have been consistently investigated in the past few years as techniques to tune API formation; however, combining these methods can be used to provide a system for rational design of drug polymorph formation and drug crystal size and habit. A system that allows control over the surface and the space available for crystallization enables constriction of particles within the confined pores to induce nucleation with surface functional groups directing specific interactions to control the API polymorph. Recently, researchers have investigated these two techniques together in order to understand the fundamental effects of combined crystallization methods on API polymorphism and the final drug crystal products [78,104,105]. In order to combine the effects of confinement and surface templating, porous material with specific chemistries are required. Therefore, platforms typical for this are cross-linked polymer networks, physical gels, and porous particles of small organic materials.

### 4.1. Crystallization in Cross-Linked Polymer Networks

Cross-linked polymers are entangled, chemically cross-linked networks where the molecular weight of the polymer can be used to manipulate the mesh size. These polymer systems can be used as films and microspheres to crystallize APIs. The functional groups within the polymers are used to form interactions with APIs and confinement within the mesh determines the crystal size and shape while also inducing nucleation.

Diao et al. used cross-linked polymers to crystallize aspirin (ASP) and showed the dependence of ASP crystal face on the surface functional groups. The (011) face of ASP, which is rich in carboxyl groups, preferentially interacted with the tertiary amide functionalities on poly(4-acryloylmorpholine) substrates and the carbonyl rich (100) face of aspirin interacted with poly(2-carboxyethyl acrylate) surfaces, which contain a high number of carboxyl groups. Additionally, the pores on these surfaces significantly decreased the time required for nucleation of aspirin crystals due to induced supersaturation enabling a much higher number of crystals to form. The surface chemistry was used to direct the ASP polymorph and confinement helped induce crystallization [78].

**Figure 5:**
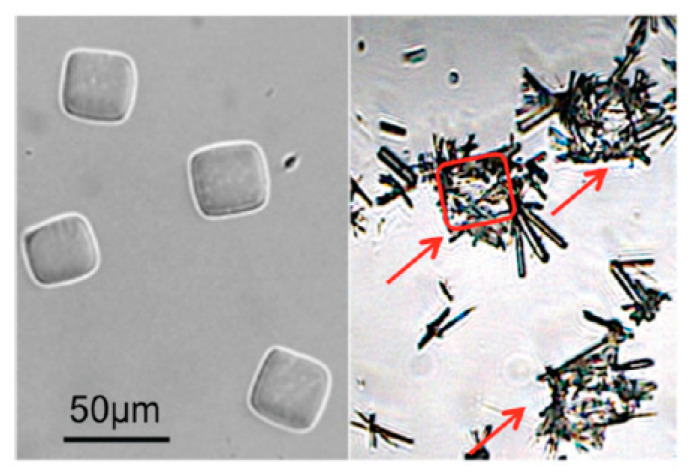
Optical micrographs of PEG400DA microgels as synthesized (**left**) and CBZ metastable form II needles grown on microgels (**right**). 3 microgels covered with CBZ needles are indicated with red arrows and the contour of the middle one is traced with a red line to delineate the cubic gel. Reprinted with permission from [107], the American Chemical Society, 2012.

The same group also investigated the use of cross-linked polyethylene glycol diacrylate (PEG-DA) microgels to crystallize ASP and acetaminophen (ACT). Both of these drugs preferentially crystallized onto or within the particles. The surface chemistry of the microgel enabled hydrogen bonding with the drug crystals and the confinement restricted the motion of the drug crystals to facilitate the favorable interactions between the APIs and the polymer matrix [106]. Diao et al. further expanded this research to study the nucleation of CBZ and ROY in PEG-DA microgels. They found that the metastable form II of CBZ could be crystallized within the polymer mesh due to enhanced nucleation kinetics and by forming interactions between the vinylic hydrogen of CBZ and the oxygens on the PEG chains (Figure 5). While surface chemistry enabled the hydrogen bonding interactions, this alone did not result in CBZ form II. Confinement was also essential to nucleate the metastable form due to the lowered energy barrier from supersaturation within the microgel. Additionally, the PEG-DA microgel promoted the nucleation of ROY metastable form R due to hydrogen bonding between the amine hydrogen of ROY with the carbonyl oxygen of PEG-DA. Again, while this was primarily attributed to surface chemistry, the mesh size of the microgel also had an effect on polymorphism. It was only at optimum mesh sizes where the confinement balanced the solute-solute interactions with the polymer-solute interaction to enable nucleation from the polymer surface and not the bulk [107].

### 4.2. Crystallization in Physical Gels

The use of gels in the pharmaceutical field has been studied by several groups, especially for encapsulation and drug delivery applications since gels can be swelled in certain media for the easy release of entrapped drug crystals [108,109,110]. More recently, crystallization of APIs within gel networks has gained traction due to control of over both surface geometry and surface chemistry [105,111,112]. While some gels are a type of cross-linked polymer networks, they can also be formed using lower molecular weight organics and even inorganic materials that attract through physical interactions. Gels typically contain pores with small mesh sizes, which can play a dramatic role in enhancing nucleation kinetics by forcing supersaturation [112,113,114], and the surface of the pores within the gel can be modified to enable binding of specific API functional groups and presenting sites for heterogeneous nucleation of drug crystals [104,105]. Additionally, the solute-solvent interaction is an important benefit for crystallization systems involving gels [104].

Pauchet et al. used a tetramethoxysilane (TMOS) gel to crystallize ± modafinil (MOD), a stimulant drug used to treat narcolepsy. The form of MOD could be controlled using different solvents present in the gel and, while most systems presented with stable form I, nucleation of the metastable form III was achieved using a 2:7:7 ratio of TMOS:H_2_O:methanol. The surface of the gel initiated the original crystal growth and confinement in gels allowed crystal growth via stacking of individual planes since the gel mesh limited external interactions [104]. Aparicio et al. used organogelators to crystallize CBZ from toluene. Homogeneous nucleation from toluene and crystallization using an achiral gelator resulted in the stable form III; however, the addition of a chiral gelator resulted in the heteronucleation of form II. They ascribed this to the difference in morphology of the gel depending on amount of the chiral gelator used [115].

**Figure 6:**
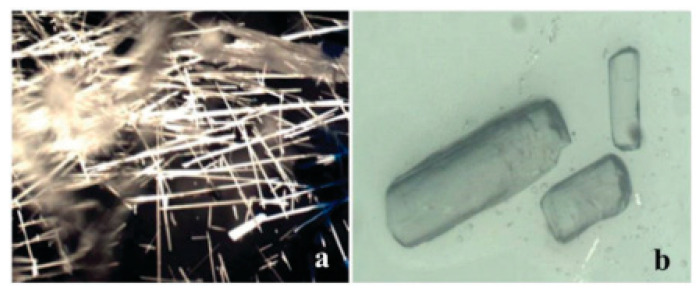
(**a**) Needle shaped crystals of isoniazid grown from DMSO solution and (**b**) block shaped crystals from DMSO ODA/C-CNC gels under the same conditions [116]. Reproduced from [116], The Royal Society of Chemistry, 2016.

Palomero et al. used organogels of carboxylated cellulose nanocrystals (C-CNCs) and octadecylamine (ODA) to crystallize several different drugs using DMSO as the solvent. They showed the formation of solvates with DMSO for both sulfapyridine (SP) and sulfamethoxazole (SMX) within the C-CNC gel network. Solvates are forms of a drug where solvent molecules are trapped within the crystal structure. They further showed that the C-CNC gels were able to change the crystal habit of the tuberculosis drug, isoniazid (INZ) from thin needles to block-shaped (Figure 6). The C-CNC gel surface chemistry caused specific interactions to direct the INZ crystal habit change and the confinement within the gels induced nucleation of the SP and SMX solvate forms, which are not obtained from bulk solution crystallization [116].

In our lab, we expanded on this work and used similar gels of C-CNC and ODA in DMSO for the solidification of SMX, SP, and sulfamerazine (SMZ). We showed that SMX was recovered from the gel network as an amorphous form, while crystallization out of DMSO resulted in a solvate form. This was attributed to confinement suppressing the formation of nuclei. The crystal structure of SP in the gel was a combination of forms I, III, and IV (compared to form I for as-received) and the crystal habit of SMZ was changed from needle-like to plate-like. The changes for both SP and SMZ were attributed to a combination of surface chemistry effects, resulting in changing the polymorphs and crystal habit, as well as confinement leading to inducing crystallization and trapping the crystal habit [117]. These studies showed the ability of these C-CNC gels to change the polymorphic forms of APIs as well provide control over the drug crystal habit.

**Figure 7:**
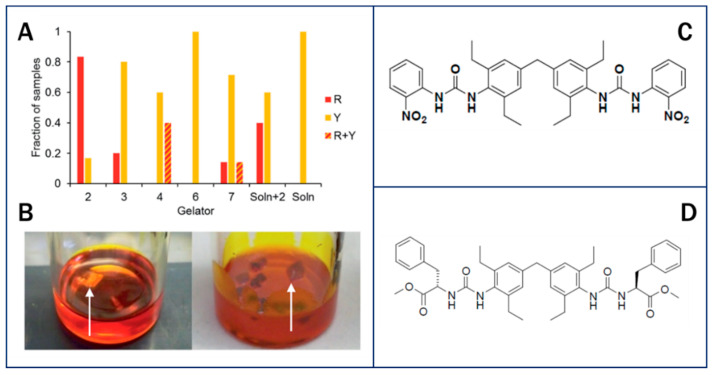
(**A**) Collated data comparing the form of ROY obtained from 100 mg/mL toluene gels of designer gelator 2, non-specific gelators 3, 4, 6 and 7, from toluene solution saturated with 2 and from solution. (**B**) Crystallization of the Y form of ROY from a toluene gel of control compound 7 (left) and the R form from a toluene gel of 2 (right). (**C**) ROY-Specific bis(urea) gelator molecule 2. (**D**) Non-specific L-phenylalanine derivative gelator 7 [118]. Reproduced and modified from [118], The Royal Society of Chemistry, 2017.

Foster et al. studied the crystallization of ROY within organogels using bis(urea) gelators with similar structures to the ROY molecule. These gels were able to crystallize the triclinic metastable red (R) form of ROY, while non-specific gelators formed the stable monoclinic yellow (Y) polymorph (Figure 7). The surface chemistry of the urea groups in the specific gels provided good electrostatic matching with the thiophene group in form R. Additionally, confinement within the gel resulted in reduced flexibility of the molecules, which resulted in stronger interactions between gelators and API, and enabled the direct the formation of the R polymorph of ROY [118].

Foster et al. further used low-molecular-weight gelators to crystallize CBZ. They showed that, when using gels, they were able to crystallize the metastable form II of CBZ and stabilize the drug, whereas without the gel, form II transitioned to stable form III in solution. Additionally, they showed that that the fibers of the gels trap the needle-shaped crystals and instead of further crystal growth, nucleation of “daughter crystals” from a parent crystal was observed. While the surface chemistry resulted in the nucleation of form II, confinement led to the changes in crystal habits and controlled the overall crystal morphology [112].

### 4.3. Porous Particles of Organic Small Molecules

Agglomerated particles, such as crystals of small organic molecules, can also be used as templates for API crystallization [72]. Heterogeneous crystallization from such materials has already been discussed but agglomerates can also present with surface roughness, where features act as micropores and have the further advantage of confinement. The differences in surface morphology result in access to crystal faces that would otherwise not be possible [104,111], which results in additional control over the crystallization process compared to using a flatter surface such as a film or single crystal.

Quon et al. studied the nucleation of ACT on spherical agglomerates of triclinic lactose crystals and found that the agglomerates enhanced the nucleation kinetics of ACT by a factor of 11 in comparison to single lactose crystals. Pores between the agglomerates led to increased surface area for crystallization and favorable energy interactions were achieved due to the coincident lattice matching between the [141] face of lactose and the [001] face of ACT. The [141] crystal plane was not observed on single crystals of lactose indicating that the morphology change from agglomeration resulted in this new possibility for lattice matching, which shows the benefit of combined effects of surface chemistry and confinement for crystallization [72].

Ling and Chadwick used microporous particles of alginate and carboxymethyl cellulose to directly crystallize acetaminophen (ACT) and sulfathiazole (STZ) via favorable surface interactions. The extent of crystal growth was determined by the free volume available inside the pores of the particles, allowing control over particle size. While surface chemistry determined the API polymorph, the crystal size was controlled by confinement. They further showed that drug encapsulation efficiency was higher with direct crystallization, indicating that combining confinement within porous surfaces with specific surface chemistries can successfully be used for particle engineering [77].

## 5. Use of Surface Chemistry and Confinement Outside of Pharmaceuticals

Several areas outside of pharmaceuticals have made use of the advantage of surface chemistry and/or confinement control to engineer crystals of a wide variety of materials including organic semiconductors (OSCs), proteins, inorganic compounds, and even explosives. OSCs are carbon-based conductive materials that are highly sought after due to being light-weight and flexible while offering the properties of inorganic semiconductors [119]. Gao et al. used bisurea-based organogels to obtain single crystals of a variety of OSCs. While this study only preliminarily addressed crystallization, they showed the ability to control the molecular aggregation of the gels based on concentration of the gelator, which could be used to control the morphology and habits of the crystals [120]. Liu et al. nucleated OSC single crystals from templates of single-walled carbon nanotubes (SWNTs). The π-π interactions between the SWNTs and the aromatic OSCs direct the crystallization and the roughness of the SWNT bundles amplifies these effects, leading to high nucleation density. Liu et al. suggested that this combined approach was essential for patterning OSC single crystals [121].

Agarose gels were used by Wang and Liu to nucleate lysozyme protein crystals. These gels were shown to inhibit diffusion of the protein nuclei due to the gel mesh network. Additionally the structure mismatch between gel surface and proteins reduced the rearrangement of the proteins and led to larger protein crystals with fewer defects [122]. Tanabe et al. further studied agarose gels for nucleating lysozymes and found that these gels were able to increase nucleation density by 10-fold. They posited that the porous nature of the gels enhanced supersaturation and allowed for electrostatic interactions between the protein and the agarose surface, which promoted heterogeneous nucleation [123]. This is similar to research performed by Chayen et al., which studied the use of mesoporous bioactive gel-glass to nucleate various proteins, including lysozyme, thaumatin, and trypsin. Their study showed that the porosity in conjunction with electrostatic interactions between the protein and the gel-glass surface charges resulted in inducing nucleation of the proteins at metastable conditions and obtaining well-formed protein crystals [124].

Stack et al. utilized unmodified and functionalized controlled glass pores (CPGs) to crystallize calcium carbonate within the pores. In unmodified CPGs, crystallization was only induced in macropores, whereas CPGs coated with 3-(triethoxysilyl)propylsuccinic anhydride were able to induce CaCO_3_ crystallization into the nanopores due to favorable interactions with the pore walls [125].

## 6. Summary and Outlook

The studies above show the potential for using a combined technique of confinement and surface templating in order to direct crystallization of APIs, but it is clear that the approach is still in its infancy. The benefits of using each technique individually have been well-documented and show outstanding promise. Through the studies discussed in Section 4, it is clear that, when combining these two crystallization avenues, it is possible to use the advantages of both techniques in order to rationally design systems for API nucleation, especially when polymorph selectivity is of importance. While the studies that focus on examining both approaches together are few, only the 13 discussed in Section 4, a careful examination of many of the works in Section 3 show that evidence for complementary effects have existed for some time. In several studies that aimed to use confinement to control crystallization, they noted that the solvent or surface functionality [53,96] played a role in the crystal properties, indicating that molecular interactions between the crystallizing drug and the surface were also influencing the results, if not the primary design focus. Similarly, in studies on heterogenous nucleation from designed surfaces, surface roughness and size [79,91,93] as well as functional group density [69] had an effect on the drug crystals. This showed that the morphological surface characteristics, though not always confinement, influenced the supersaturation and thus the crystal that formed.

Overall, using design approaches that combine surface functionality (and thus molecular interactions between API and surface) with confinement allow for more tunability of the API crystal that forms. Importantly, even for single-approach designs, a consideration should be made for both, as the surface and chemical functionalities will always impact the molecular arrangement of crystallizing drug molecules and the pore size or even surface roughness will impact local supersaturation and crystal size. Thus, further fundamental work on the combined approaches will have far-reaching impacts on all types of crystal engineering design approaches.

These dual-approach systems are versatile as seen from the examples above. In the pharmaceutical industry, an especially strong advantage is that they can be more flexible than designing new surfaces for each newly developed drug product. The use of directed crystallization is appealing from a commercial standpoint as it helps in developing drug crystals with better efficacy and bioavailability [114]. By carefully selecting biocompatible templates for confinement and nucleation, it is also possible to develop composite materials of API and surfaces, eliminating the need for additional excipients or stabilizers and this is especially true for gel-based systems, which are stable and allow for easy handling. However, additional research needs to be done before these systems can be successfully utilized in the drug discovery and development process. Currently, only a handful of small molecule APIs have been tested in combined crystallization systems and most research is still limited to drugs that have been well studied and have established production methods. Expanding the field of study to more types of drugs is required before this method can be tested in industry.

Additionally, as continuous crystallization becomes more prevalent, studies into implementing combined crystallization systems into continuous crystallizers will be valuable. Preliminary studies of adding excipients into continuous crystallizers to promote heterogeneous nucleation onto a surface has been done by a few groups, especially at the Novartis-MIT Center for Continuous Manufacturing [67,126,127] and EPSRC CMAC Future Manufacturing Research Hub [128,129]. These studies point to the possibility of introducing gels or other combined crystallization systems into continuous crystallization setups such as mixed suspension mixed product removal crystallizers. Microgels or microporous organic particles could be included in a continuous crystallizer similar to the D-mannitol excipients used by Yazdanpanah et al. [67], however further research into the implementing these combined systems needs to be carried out. Finally, while some surface induced crystallization is already used in industry, testing the viability of a combined approach requires additional studies into scalability. These considerations have to be taken into account before combined methods of crystallization can be applied into the pharmaceutical industry.
